# Development and validation of an MRI-based radiomic model for predicting overall survival in nasopharyngeal carcinoma patients with local residual tumors after intensity-modulated radiotherapy

**DOI:** 10.1186/s12880-022-00902-6

**Published:** 2022-10-04

**Authors:** Shengping Jiang, Lin Han, Leifeng Liang, Liling Long

**Affiliations:** 1 Department of Radiology, The First Affiliated Hospital of Guangxi Medical University, No. 6 Shuangyong Road, Nanning, 530021 China; 2 Department of Rehabilitation Medicine, The First People’s Hospital of Yulin, No. 495 Jiaoyu Road, Yulin, 537000 China; 3 Department of Radiation Oncology, The First People’s Hospital of Yulin, No. 495 Jiaoyu Road, Yulin, 537000 China; 4 Key Laboratory of Early Prevention and Treatment for Regional High Frequency Tumor (Guangxi Medical University), Ministry of Education, Nanning, Guangxi China

**Keywords:** Nasopharyngeal carcinoma, Residual tumor, MRI, Radiomics

## Abstract

**Background:**

To investigate the potential value of the pretreatment MRI-based radiomic model in predicting the overall survival (OS) of nasopharyngeal carcinoma (NPC) patients with local residual tumors after intensity-modulated radiotherapy (IMRT).

**Methods:**

A total of 218 consecutive nonmetastatic NPC patients with local residual tumors after IMRT [training cohort (n = 173) and validation cohort (n = 45)] were retrospectively included in this study. Clinical and MRI data were obtained. Univariate Cox regression and the least absolute shrinkage and selection operator (LASSO) were used to select the radiomic features from pretreatment MRI. The clinical, radiomic, and combined models for predicting OS were constructed. The models’ performances were evaluated using Harrell’s concordance index (C-index), calibration curve, and decision curve analysis.

**Results:**

The C-index of the radiomic model was higher than that of the clinical model, with the C-index of 0.788 (95% CI 0.724–0.852) versus 0.672 (95% CI 0.599–0.745) in the training cohort and 0.753 (95% CI 0.604–0.902) versus 0.634 (95% CI 0.593–0.675) in the validation cohort. Calibration curves showed good agreement between the radiomic model-predicted probability of 2- and 3-year OS and the actual observed probability in the training and validation groups. Decision curve analysis showed that the radiomic model had higher clinical usefulness than the clinical model. The discrimination of the combined model improved significantly in the training cohort (*P* < 0.01) but not in the validation cohort, with the C-index of 0.834 and 0.734, respectively. The radiomic model divided patients into high- and low-risk groups with a significant difference in OS in both the training and validation cohorts.

**Conclusions:**

Pretreatment MRI-based radiomic model may improve OS prediction in NPC patients with local residual tumors after IMRT and may assist in clinical decision-making.

**Supplementary Information:**

The online version contains supplementary material available at 10.1186/s12880-022-00902-6.

## Introduction

Nasopharyngeal carcinoma (NPC) is a carcinoma originating from the epithelium of the nasopharyngeal mucosa with unique clinical features. The incidence of nasopharyngeal cancer differs by geographical distribution and across racial populations and is particularly prevalent in some East and Southeast Asian countries and regions. According to GLOBOCAN (2020), there were about 133,354 new cases of nasopharyngeal cancer worldwide and about 80,008 new deaths from nasopharyngeal cancer in 2020 [1]. With the progress of diagnostic imaging techniques for NPC, the development of comprehensive treatment strategies, especially the widespread use of intensity-modulated radiotherapy (IMRT), and the change in people's lifestyles [2], the mortality of NPC has shown a downward trend in most countries and regions all over the world [3, 4]. As the primary treatment modality for NPC, IMRT increases the conformity of tumor coverage while reducing radiation damage to surrounding tissues [5]. Nevertheless, there are still a considerable number of patients with local residual tumors after radiation therapy [6]. Ping-Yan Liao et al. reported that approximately 27.3% of patients (147 out of 538) with nonmetastatic NPC had local tumor residuals within 6 months after radiotherapy [7]. Patients with NPC who have residual tumors after radiotherapy are more likely to have a worse prognosis [8]. The latest CSCO and ASCO joint guideline on definitive-intent chemoradiotherapy for patients with stage II-IVA NPC recommends an additional 2–4 Gy boost as one of the management modalities for patients with MRI-detected residual tumors after IMRT [8]. However, the toxic burden is also an issue that needs attention. Identifying those with poor prognosis among patients with residual tumors after IMRT may facilitate subsequent more personalized treatment measures to improve their prognosis.

In terms of the detection of residual NPC after radiotherapy, the majority of residual tumors are located outside the nasopharyngeal cavity [6], making pathological confirmation via biopsy challenging to obtain. Furthermore, 26.4% of residual lesions in the nasopharynx are missing from single biopsies [9]. Research by Maurizio Comoretto et al. has demonstrated that MR imaging tends to have higher accuracy than FDG-PET-CT in detecting residual and/or recurrent NPC at the primary site after radiotherapy (92.1% vs. 85.7%) [10]. The study by Shu-Hang Ng et al. has revealed that the diagnostic performance of MRI for residual/recurrent NPC was similar to that of FDG-PET-CT [11]. Considering the high cost and radiation exposure of FDG-PET-CT, MRI is the optimal modality for the assessment of residual NPC after treatment and is also consistent with the guideline described above [8].

Radiomics involves the high-throughput extraction of a large number of features from radiographic images [12], converting radiographic images into data that can be mined by bioinformatics tools to develop diagnostic, predictive, and prognostic models [13]. Several recent studies have explored the potential value of pretreatment MRI-based radiomics in response to induction chemotherapy, progression-free survival for NPC. Wang et al. used the radiomics signature established from 15 features extracted from pretreatment T1WI, T2WI, T2WI with fat suppression, and CE T1WI sequences to predict the response of induction chemotherapy in NPC patients with an AUC of 0.822 [14]. Shen and colleagues investigated the role of MRI-based radiomics in predicting PFS in non-metastatic NPC patients and found that the radiomic model performed better than the clinical data model, with the C-index of 0.749 versus 0.563 in the training group and 0.836 versus 0.456 in the validation groups [15]. Zhang et al. have investigated the role of radiomic features of multiparametric MRI in predicting PFS in advanced NPC, and the C-index of the radiomic signature from joint contrast-enhanced T1WI (CE-T1WI) and T2WI images was 0.758 and 0.737 in the training and validation cohorts, respectively [16]. However, the role of MRI-based radiomics in predicting the overall survival (OS) of NPC patients with local residual tumors following IMRT has not been studied.

In the current study, we developed and validated an MRI-based radiomic model to predict OS in nonmetastatic NPC patients with local residual tumors after IMRT and stratify patients into different risk groups using the radiomic model. The tumor volumes are often retracted to varying degrees after IMRT, which may affect the robustness of the radiomic features [17]. In addition, a variety of changes occurred after radiotherapy, making the composition of the residual tumor more complex than before treatment. We chose to extract radiomic features from pre-treatment MRI to build the model.

## Patients and methods

### Study population

The Medical Ethics Committee of First Affiliated Hospital of Guangxi Medical University approved the study (NO. 2022-KY-E-249), and the informed consent of the patients was waived for the nature of the retrospective study.

The study cohort was from an endemic region where nonkeratinizing pathological subtype accounts for more than 95% of the cases [18]. We retrospectively reviewed 925 nonmetastatic patients with NPC who received IMRT in our hospital from May 2015 to December 2017. Participants were selected according to the inclusion and exclusion criteria below. Inclusion criteria included the following: (1) NPC was pathologically confirmed; (2) the initial MRI scans were completed prior to treatment, and there was no more than 1 week between receiving treatment and the initial MRI scans; (3) local residual tumors were diagnosed immediately after the completion of IMRT. The exclusion criteria were as follows: (1) the patients had been treated in other hospitals prior to their admission to our hospital; (2) those who were unable to complete the radiotherapy course; (3) those whose MRI images were marred by artifacts caused by patient movement, oral metal materials, or other factors; (4) patients with other malignant tumors or NPC with distant metastasis; and (5) those lost to follow-up (with a loss to follow-up rate of 4.4%). Finally, 218 eligible NPC patients with local residual disease after IMRT were enrolled in this study, and the patients were randomly divided into training and validation cohorts according to the ratio of 8:2. Demographic characteristics of the study population, the American Joint Committee on Cancer (AJCC) staging of NPC, pretreatment hemoglobin level, neutrophil to lymphocyte ratio (NLR), albumin level, as well as chemotherapy strategies (induction chemotherapy, concurrent chemotherapy, adjuvant chemotherapy) were collected. The tumors were restaged in accordance with the eighth edition of the AJCC staging manual.

### Treatment strategy and follow-up

The treatment of NPC is carried out under the relevant guidelines: definitive radiotherapy was applied to patients with T1N0M0; concurrent chemoradiotherapy followed or not followed by adjuvant chemotherapy or induction chemotherapy followed by chemoradiotherapy for patients with T1, N1-3, M0, and T2–T4, any N, M0 [19, 20]. All patients completed the course of IMRT. Details on the radiotherapy protocol are included in Additional file 1. Platinum-based regimens were used for chemotherapy. During the first 3 years following IMRT, patients were followed-up every 3 months, every 6 months during the fourth and fifth years, and once a year after the fifth year. OS, the endpoint of our study, was defined as the time from the completion of IMRT to the date of death or last follow-up. The patient's survival status was ascertained through telephone consultation with the patient or his family. Living patients were censored between July and August 2021.

### MR Imaging Protocol

All MRI scanning of the patients was performed on a GE Signa EXCITE 1.5 T MRI Scanner (GE Medical Systems, USA). Scanning sequences included axial fast relaxation fast spin echo (FRFSE) T2 weighted imaging (T2WI) sequence, axial/sagittal/coronal unenhanced T1WI Flair sequences, and axial/sagittal/coronal CE-T1WI Flair sequences with fat suppression. Gadopentetate Dimeglumine Injection (Bayer Healthcare Pharmaceuticals Inc.) was used as the contrast agent at a dose of 0.2 ml/kg body weight (up to 10 ml) and an injection rate of 2 ml/s. Radiomic features were extracted from pretreatment axial T2WI and CE-T1WI sequences. The main acquisition parameters of axial FRFSE T2WI sequence included: repetition time (TR) = 2620 ms, echo time (TE) = 101 ms, field of view (FOV) = 220 mm × 220 mm, matrix = 320 × 160, flip angle = 90°, slice thickness = 6 mm, intersection gap = 1 mm. The main acquisition parameters of axial CE-T1WI sequence included: TR = 2162 ms, TE = 10 ms, FOV = 220 mm × 220 mm, matrix = 320 × 160, flip angle = 90°, slice thickness = 6 mm, intersection gap = 1 mm.

### Diagnostic criteria of local residual tumors

**Fig. 1:**
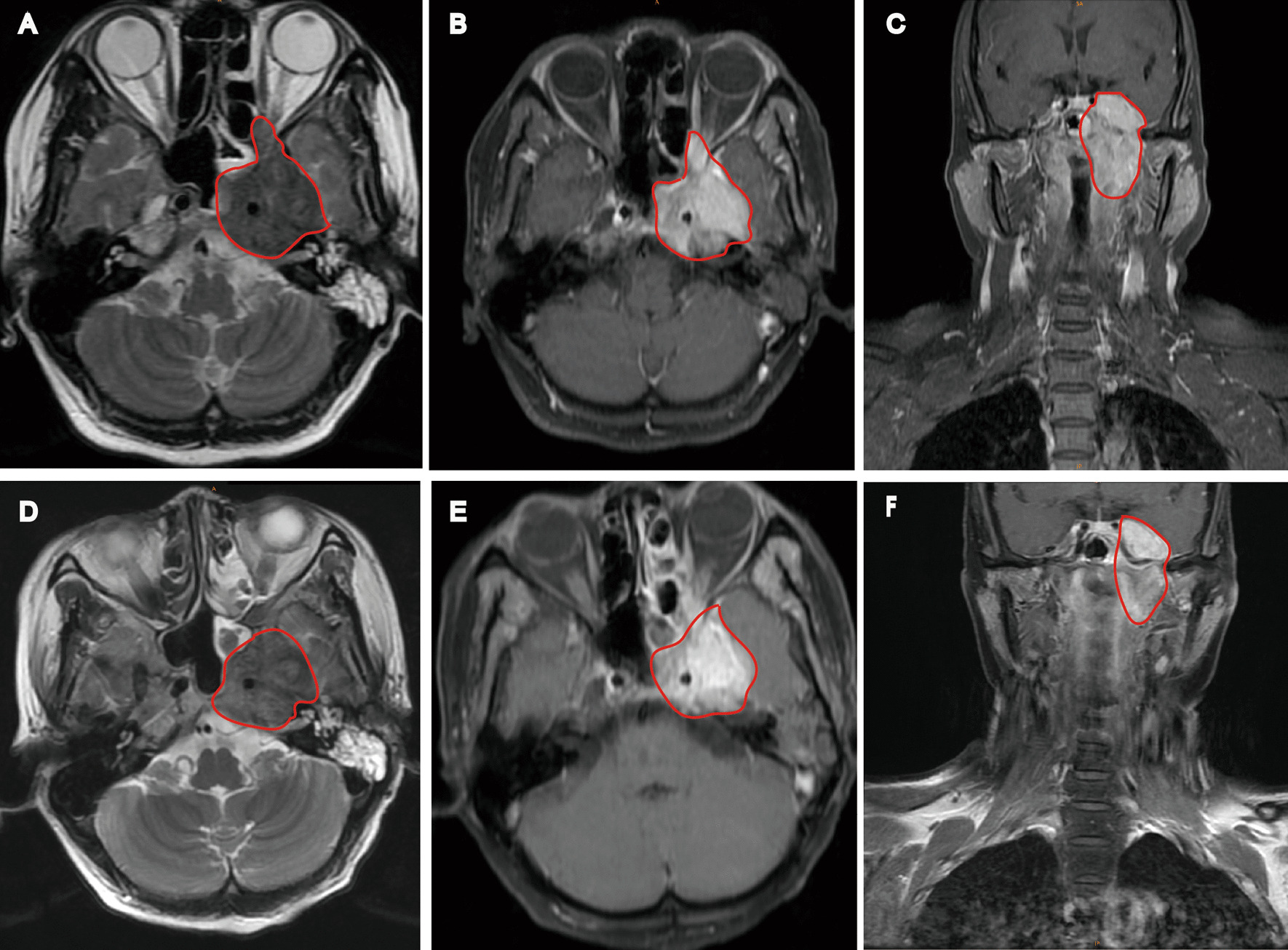
Pre-and post-treatment MRI of a 42-year-old female nasopharyngeal carcinoma patient with local residual disease after intensity-modulated radiation therapy. Pre-treatment MRI is shown in **A**–**C**; post-treatment MRI is shown in **D**–**F**. Post-treatment MRI indicated a local residual tumor in the nasopharynx, skull base, and intracranial space

Post-treatment MRI scanning was performed immediately after the completion of the course of IMRT to assess tumor regression. Blind to the clinical outcomes of the patients, a head and neck oncology radiologist (Investigator A) with 9-year experience and a head and neck radiation oncologist with 14-year experience (Investigator B) evaluated the MR images, respectively. The divergence between the two investigators regarding the evaluation of the MR images will be adjudicated through a third head and neck oncology radiologist (Investigator C) with 30-year experience to achieve agreement. The diagnostic criteria of local residual tumors on MRI were as follows: (1) residual tumors in the nasopharynx, parapharyngeal soft tissues, or intracranial spaces exhibit low signal intensity on T1WI, intermediate signal intensity over muscle on T2WI, and moderate signal enhancement on CE-T1WI [21]; (2) lesions of the skull base were thought to be tumor residuals if the bone of the skull base was destroyed by soft tissues and the degree, and/or extent of bone strengthening was not diminished or increased compared with pretreatment images [11, 22]. Figure 1 shows MRI images of the local residual tumor in a patient following IMRT.

### Lesions segmentation and radiomic features extraction

**Fig. 2:**
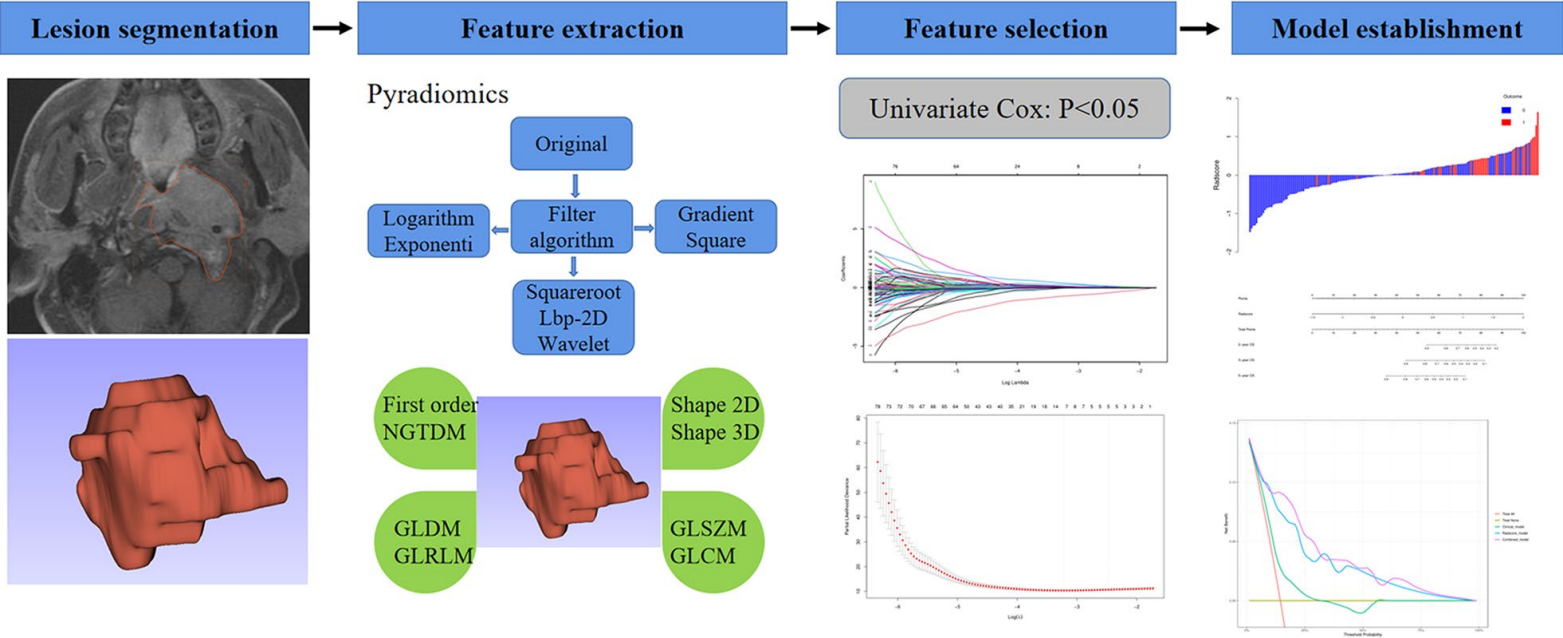
The workflow of radiomics analysis

The workflow of radiomics analysis is shown in Fig. 2. The delineation of the regions of interest (ROI) and features extraction were carried out on the RadCloud Radiomics Cloud Platform (Huiying Medical Technology Co., Ltd) using pretreatment T2WI and CE-T1WI sequences of the nasopharynx. Radiomic features extraction was based on pyradiomics 2.2.0.

We randomly selected 30 patients from the study population to assess the inter-and intra-class correlation coefficients (ICCs). Firstly, each of the 218 patients was assigned a number between 1 and 218; after that, 30 of those numbers were chosen at random. Blind to the clinical outcomes of the patients, Investigator A and Investigator B delineated the ROI of the entire tumor layer by layer in the patient's nasopharyngeal T2WI and CE-T1WI images independently. Investigator A repeated the process 2 weeks later. Before feature extraction, images were resampled using the B-spline interpolator to a pixel spacing of 1.0 mm × 1.0 mm × 1.0 mm and normalized based on the mean and standard deviation of gray values.

1409 features for each patient and each sequence were extracted from the original images (107 features) and derived images using filters including logarithm (93 features), exponential (93 features), gradient (93 features), square (93 features), squareroot (93 features), localBinaryPattern2D (lbp-2D, 93 features), and wavelet (744 features). These 1409 features included first order statistics (270 features), Shape-based (14 features), Neighbouring Gray Tone Difference Matrix (NGTDM, 75 features), Gray Level Co-occurrence Matrix (GLCM, 360 features), Gray Level Dependence Matrix (GLDM, 210 features), Gray Level Run Length Matrix (GLRLM, 240 features), and Gray Level Size Zone Matrix (GLSZM, 240 features). A total of 2818 features were extracted from each patient's images. Most of the features extracted conform to IBSI definitions of features. The pyradiomics documentation provides explanations of these radiomic features (https://pyradiomics.readthedocs.io/en/latest/index.html). Z-score normalization of the features was performed to eliminate the effect of extreme values and facilitate the comparison of features of different magnitudes. Features with ICCs > 0.75 reveal good reproducibility and will be used for further analysis. Subsequently, Investigator A conducted lesion segmentation and feature extraction on the remaining samples.

### Selection of radiomic features and establishment of radiomic signature

Firstly, the features with ICCs > 0.75 were tested for the proportional hazards assumption in the training cohort, and then the features that met the proportional hazards assumption were included in the univariate Cox regression analysis. The least absolute shrinkage and selection operator (LASSO)-Cox regression [23] based on statistically significant features with a *p*-value < 0.05 of the univariate Cox regression analysis was conducted. The optimal lambda was selected based on tenfold cross-validation. Features with non-zero coefficients were selected to establish the radiomic signature. Radscore is calculated based on the value of the features and their coefficients multiplied together.

### Statistical analysis and modelling

Continuous variables are expressed as the means and standard deviations if they are normally distributed; otherwise, they are expressed as the medians and interquartile ranges. Categorical data are expressed as frequencies and percentages. Independent sample t-tests or Mann–Whitney U tests were used to compare continuous variables between the training and validation cohorts. χ^2^ or Fisher exact tests were used for the comparison of categorical variables between the two groups. The clinical model for predicting OS was established using the independent clinical predictors identified by multivariate Cox regression in the training cohort. The radiomic model was established using radscore-based univariate Cox regression. The combined model, which integrated significant clinical factors and radscore, was constructed using stepwise back multivariate Cox regressions based on Akaike's Information Criterion (AIC). The Harrell’s concordance index (C-index) and 2-year, 3-year, and 5-year receiver operating characteristics (tROC) curves were used to evaluate the models’ discrimination. To prevent the influence of a single random training-validation set split leading to unreliable results, the discrimination of the different models was determined by fivefold cross-validation with 200 repetitions. The agreement between the model-predicted OS probability and actual observed survival rate was evaluated using calibration curves, and the clinical usefulness was evaluated based on decision curve analyses. Likelihood ratio tests for comparing nested models or the AIC were used to determine which model fit the data better in the training and validation cohorts. Kaplan–Meier method with log-rank tests was used to compare the difference in survival between high-risk and low-risk groups. All statistical analysis were conducted using SPSS 22.0 (SPSS Inc., Chicago, IL) or R 4.1 (R Project for Statistical Computing). Two-tailed *p* < 0.05 is considered statistically significant.

## Results

### Clinical data

The clinical characteristics of the patients are shown in Table 1. A total of 218 eligible patients were included in this study, including 164 males (75.2%) and 54 females (24.8%), aged 46.1 ± 11.4 years (range 16.0–81.0 years). The median follow-up time in this study was 50.4 months (range of 4.0–72.0 months). Fifty-two patients died during follow-up of the entire cohort, including 41 deaths in the training cohort and 11 deaths in the validation cohort. The 2-year, 3-year, and 5-year OS rates were approximately 91.3%, 83.5%, and 74.0%.

**Table 1:** Clinical characteristics of the patients

Characteristics	Entire cohort (n = 218)	Training cohort (n = 173)	Validation cohort (n = 45)	*P* value
Age (years)	46.1 ± 11.4	45.9 ± 11.6	46.9 ± 10.9	0.605^a^
*Gender*				0.223^b^
Male	164 (75.2%)	127 (73.4%)	37 (82.2%)	
Female	54 (24.8%)	46 (26.6%)	8 (17.8%)	
Albumin (g/L)	43.6 ± 3.5	43.8 ± 3.5	43.1 ± 3.5	0.282^a^
Hemoglobin (g/L)	135.3 ± 15.3	135.1 ± 15.2	136.2 ± 15.6	0.661^a^
NLR	2.6 (1.9–3.4)	2.5 (1.9–3.4)	2.7 (2.0–3.5)	0.356^c^
*T category*				0.575^d^
T1	8 (3.7%)	5 (2.9%)	3 (6.7%)	
T2	11 (5.0%)	10 (5.8%)	1 (2.2%)	
T3	83 (38.1%)	66 (38.2%)	17 (37.8%)	
T4	116 (53.2%)	92 (53.2%)	24 (53.3%)	
*N category*				0.643^b^
N0	17 (7.8%)	15 (8.7%)	2 (4.4%)	
N1	91 (41.7%)	71 (41.0%)	20 (44.4%)	
N2	84 (38.5%)	68 (39.3%)	16 (35.6%)	
N3	26 (11.9%)	19 (11.0%)	7 (15.6%)	
*Stage*				> 0.999^d^
I	1 (0.5%)	1 (0.6%)	0 (0%)	
II	12 (5.5%)	10 (5.8%)	2 (4.4%)	
III	78 (35.8%)	62 (35.8%)	16 (35.6%)	
IV	127 (58.3%)	100 (57.8%)	27 (60.0%)	
*Induction chemotherapy*				0.902^b^
Yes	71 (32.6%)	56 (32.4%)	15 (33.3%)	
No	147 (67.4%)	117 (67.6%)	30 (66.7%)	
*Concurrent chemotherapy*				0.159^b^
Yes	202 (92.7%)	163 (94.2%)	39 (86.7%)	
No	16 (7.3%)	10 (5.8%)	6 (13.3%)	
*Adjuvant chemotherapy*				0.751^b^
Yes	116 (53.2%)	93 (53.8%)	23 (51.1%)	
No	102 (46.8%)	80 (46.2%)	22 (48.9%)	

*NLR*, neutrophil to lymphocyte ratio

^a^*P* values calculated by the independent-samples t-test

^b^*P* values calculated by the χ^2^-test

^c^*P* values calculated by the Mann–Whitney U-test

^d^*P* values calculated by Fisher exact test

There was no significant difference in age, gender, T category, N category, stage, NLR, albumin level, hemoglobin level, with or without induction chemotherapy/concurrent chemotherapy/adjuvant chemotherapy between the training and the validation groups (*P* > 0.05).

### The clinical model for predicting OS

Age, gender, and albumin level were independent predictors of OS in the training cohort (Table 2). The clinical nomogram was constructed using these three independent predictors. The C-index of the clinical nomogram predicting OS was 0.672 (95% CI 0.599–0.745) in the training group. Cumulative/dynamic time-dependent ROC (tROC) revealed that the area under curve (tAUC) of the clinical model predicting 2-year, 3-year, and 5-year OS in the training group was 0.659 (95% CI 0.528–0.790), 0.667 (95% CI 0.572–0.763), and 0.685 (95% CI 0.572–0.798), respectively.

**Table 2:** Clinical factors predicting OS in patients with residual nasopharyngeal carcinoma after IMRT in the training cohort

Characteristics	Value	Univariate analysis		Multivariate analysis	
		HR (95% CI)	*P* value	HR (95% CI)	*P* value
*Age (years)*	45.9 ± 11.6	1.042 (1.014– 1.071)	0.003^a^		
≥ 60	20 (11.6%)	3.480 (1.738–6.966)	< 0.001^a^	2.829 (1.394–5.741)	0.004^b^
< 60	153 (88.4%)	Reference		Reference	
*Gender*					
Male	127 (73.4%)	2.884 (1.132–7.352)	0.027^a^	2.586 (1.005–6.654)	0.049^b^
Female	46 (26.6%)	Reference		Reference	
*T category*					
T1	5 (2.9%)	< 0.001 (< 0.001–NA)	0.972^a^		
T2	10 (5.8%)	1.217 (0.366–4.049)	0.749^a^		
T3	66 (38.2%)	0.788 (0.408–1.524)	0.480^a^		
T4	92 (53.2%)	Reference			
*N category*					
N0	15 (8.7%)	Reference			
N1	71 (41.0%)	5.580 (0.749–41.555)	0.093^a^		
N2	68 (39.3%)	3.519 (0.459–26.997)	0.226^a^		
N3	19 (11.0%)	6.295 (0.756–52.422)	0.089^a^		
*Stage*			0.202^a^		
I	1 (0.6%)	< 0.001 (< 0.001–NA)	0.980^a^		
II	10 (5.8%)	1.162 (0.352–3.836)	0.806^a^		
III	62 (35.8%)	0.611 (0.303–1.233)	0.169^a^		
IV	100 (57.8%)	Reference			
*NLR*	2.5 (1.9–3.4)	1.114 (0.918–1.352)	0.275^a^		
≤ 2.9	107 (61.8%)	1.290 (0.668–2.491)	0.445^a^		
> 2.9	66 (38.2%)	Reference			
*Hemoglobin (g/L)*	135.1 ± 15.2	1.009 (0.987–1.031)	0.434^a^		
≤ 132.4	68 (39.3%)	Reference	0.220^a^		
> 132.4	105 (60.7%)	1.510 (0.781–2.918)			
*Albumin (g/L)*	43.8 ± 3.5	0.993 (0.906–1.089)	0.886^a^		
≤ 40.5	35 (20.2%)	2.244 (1.175–4.285)	0.012^a^	Reference	
> 40.5	138 (79.8%)	Reference		0.477 (0.248–0.917)	0.026^b^
*Induction chemotherapy*					
Yes	56 (32.4%)	1.176 (0.623–2.221)	0.617^a^		
No	117 (67.6%)	Reference			
*Concurrent chemotherapy*					
Yes	163 (94.2%)	0.607 (0.216–1.704)	0.343^a^		
No	10 (5.8%)	Reference			
*Adjuvant chemotherapy*					
Yes	93 (53.8%)	1.015 (0.549–1.876)	0.962^a^		
No	80 (46.2%)	Reference			

*NLR* neutrophil to lymphocyte ratio

The cutoffs for NLR, hemoglobin, and albumin were determined based on maximally selected test statistics

^a^*P* values tested with the univariate Cox model

^b^*P* values tested with the multivariate Cox model

**Fig. 3:**
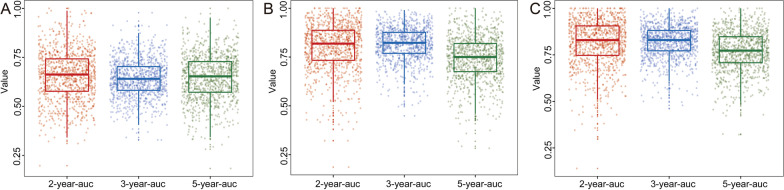
Scattered boxplots of the 2-, 3-, and 5-year mean tAUCs of the clinical model (**A**), radiomic model (**B**), and combined model (**C**) determined by fivefold cross-validation with 200 repetitions

The C-index of the clinical nomogram predicting OS was 0.634 (95% CI 0.593–0.675) in the validation group. The tAUCs of the clinical model predicting 2-year, 3-year, and 5-year OS in the validation group were 0.690 (95% CI 0.491–0.890), 0.646 (95% CI 0.488–0.804), and 0.620 (95% CI 0.398–0.841), respectively. The 2-, 3-, and 5-year mean tAUCs of the clinical model determined by fivefold cross-validation with 200 repetitions were 0.657 ± 0.126, 0.642 ± 0.094, and 0.647 ± 0.115, respectively (Fig. 3A).

### Selection of radiomic features and establishment of the radiomic signature

**Fig. 4:**
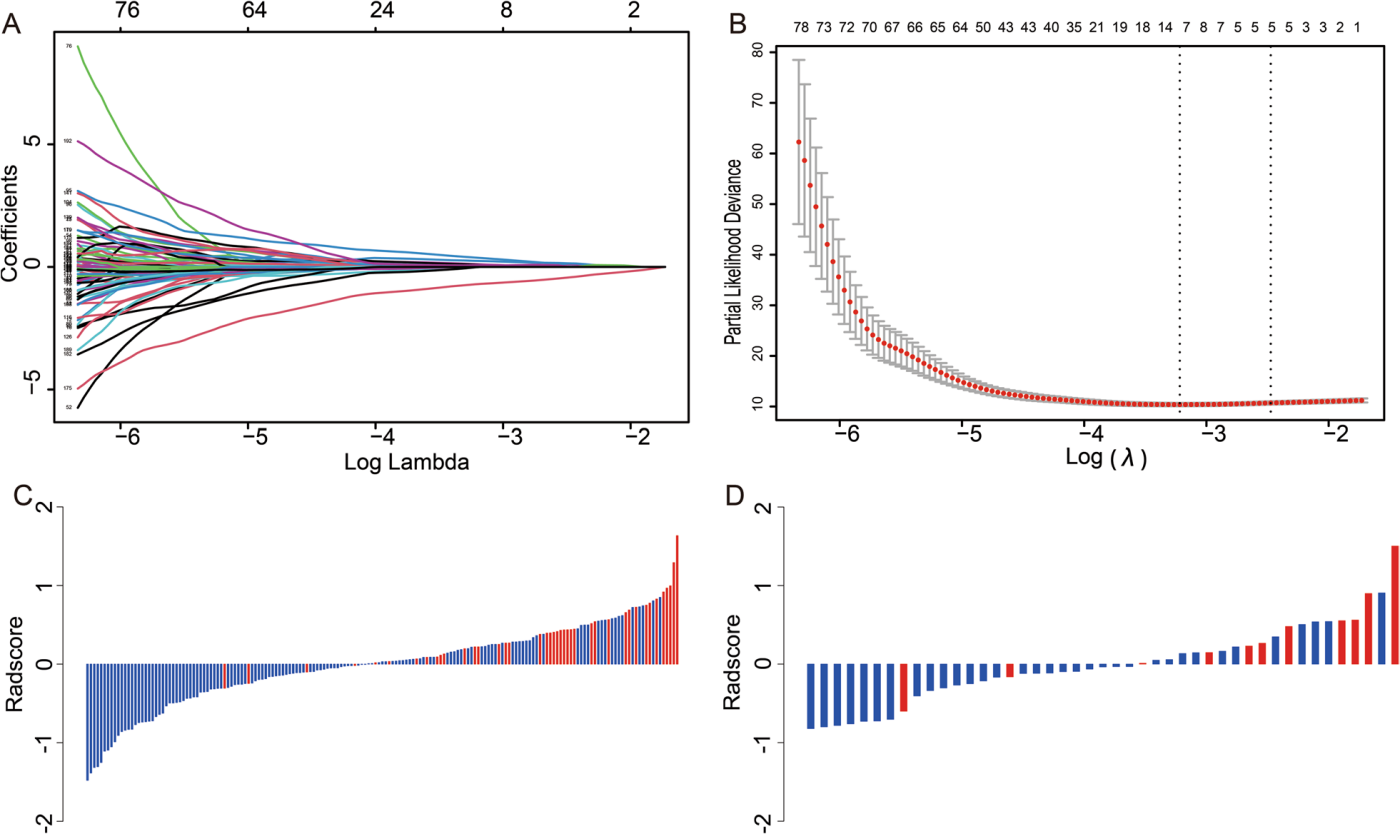
LASSO regression analysis (**A**, **B**) and radscore for patients in the training cohort (**C**) and validation cohort (**D**). **C**, **D** Each bar on the horizontal axis represents a patient. Radscore values are displayed on the vertical axis. The red bar represents the outcome of death, and the blue bar represents alive patients

Of the 2818 features extracted from T2WI and CE-T1WI sequences, 2150 features with ICCs > 0.75 were tested for the proportional hazards assumption in the training cohort. Of the features eligible for the proportional hazards assumption tests, 195 features were significant in univariate Cox regression analysis. The 195 features were then included in the LASSO-Cox regression analysis, and an optimal Lambda (λ) = 0.084 with Log (λ) = − 2.47 was selected using tenfold cross-validation via one standard error rule (Fig. 4A, B). Finally, five features with non-zero coefficients were selected. The selected features and their coefficients are shown in Table 3. The equation to calculate the radscore for each patient was as follows: Radscore = 0.081381 × T2WI_wavelet.LLL_Glrlm_ShortRunHighGrayLevelEmphasis + 0.061505 × CE-T1WI_original_Gldm_DependenceVariance + 0.142812 × CE-T1WI_original_ Ngtdm_Busyness + 0.016691 × CE-T1WI_squareroot_Firstorder_Variance—0.423306 × CE-T1WI_wavelet.LLH_Gldm_LargeDependenceLowGrayLevelEmphasis. The radscore for each patient is shown in Fig. 4C, D, and Additional file 2. The radscore of the training and validation cohorts was not significantly different (*P* = 0.903).

**Table 3:** The features selected in the radiomic model

MRI images	Feature class	Feature name	Coefficients
T2WI_wavelet.LLL	Glrlm	ShortRunHighGrayLevelEmphasis	0.081381
CE-T1WI_original	Gldm	DependenceVariance	0.061505
CE-T1WI_original	Ngtdm	Busyness	0.142812
CE-T1WI_squareroot	Firstorder	Variance	0.016691
CE-T1WI_wavelet.LLH	Gldm	LargeDependenceLowGrayLevelEmphasis	-0.423306

### Predictive performance evaluation of the radiomic model

**Fig. 5:**
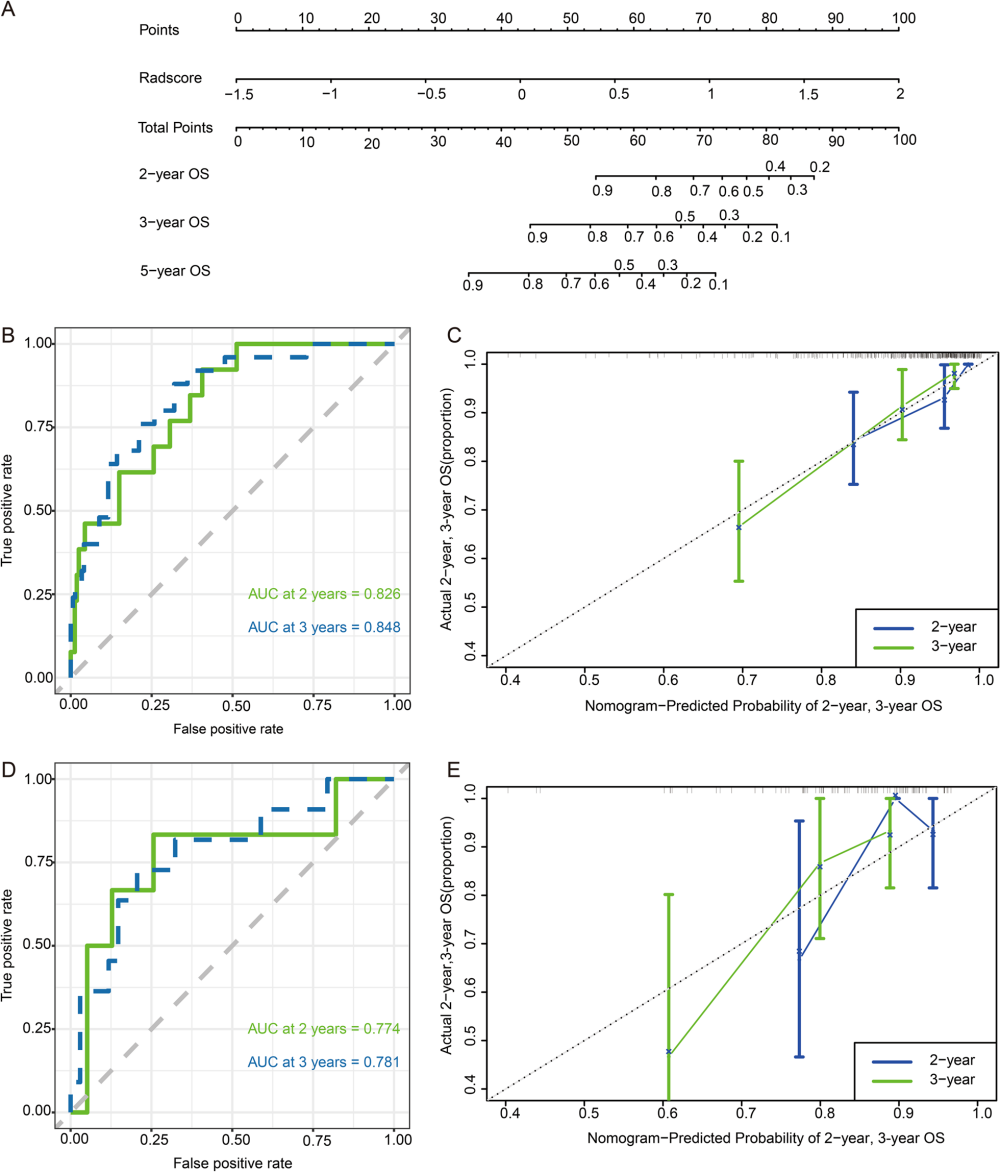
**A** The radiomic model for predicting overall survival in patients with residual nasopharyngeal carcinoma. ROC and calibration curve for predicting 2-year and 3-year OS in the training cohort (**B**, **C**) and the validation cohort (**D**, **E**)

The C-index of the radiomic model (Fig. 5) predicting OS was 0.788 (95% CI 0.724–0.852) and 0.753 (95% CI 0.604–0.902) in the training and validation cohorts, respectively. The tAUCs of the radiomic model predicting 2-year, 3-year, and 5-year OS in the training group were 0.826 (95% CI 0.727–0.925), 0.848 (95% CI 0.772–0.924), and 0.746 (95% CI 0.628–0.863), respectively, and the corresponding tAUCs in the validation group were 0.774 (95% CI 0.540–1.000), 0.781 (95% CI 0.617–0.944), and 0.719 (95% CI 0.495–0.944). The 2-, 3-, and 5-year mean tAUCs of the radiomic model determined by fivefold cross-validation with 200 repetitions were 0.802 ± 0.120, 0.817 ± 0.083, and 0.743 ± 0.113**,** respectively (Fig. 3B).

**Fig. 6:**
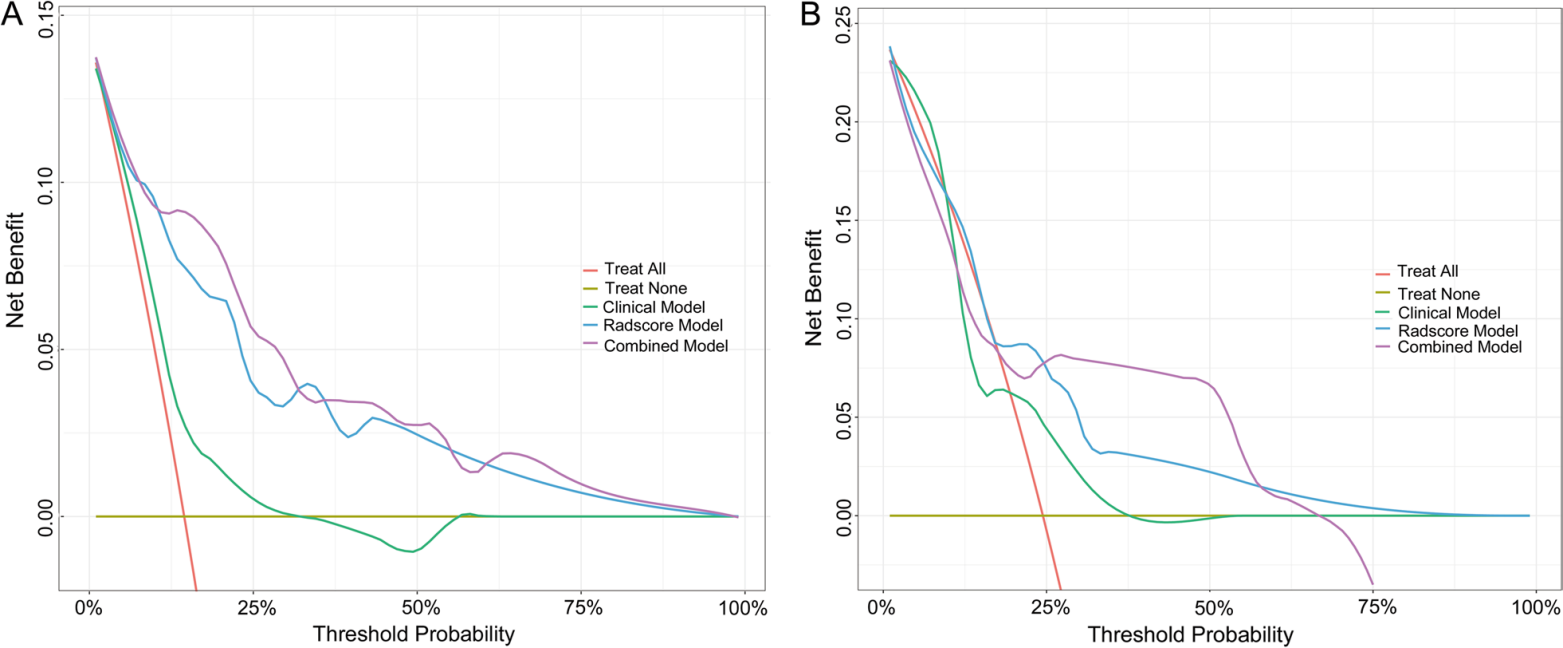
Decision curve analysis of the clinical, radiomic, and combined models for predicting 3-year overall survival in the training cohort (**A**) and validation cohort (**B**)

Calibration curves showed good agreement between the radiomic model-predicted probability of 2- and 3-year OS and the actual observed probability in the training and validation groups (Fig. 5C, E). Decision curve analysis showed that the radiomic model had higher clinical usefulness than the clinical model (Fig. 6).

### The combined model for predicting OS

Age, gender, albumin level, and radscore were ultimately included in the combined model (Additional file 3). The C-index of the combined model predicting OS was 0.834 (95% CI 0.777–0.891) and 0.734 (95% CI 0.688–0.780) in the training and validation groups, respectively. In the training group, tAUCs for predicting 2-year, 3-year, and 5-year OS were 0.848 (95% CI 0.745–0.951), 0.869 (95% CI 0.794–0.943), and 0.815 (95% CI 0.712–0.919), respectively, and the corresponding tAUCs in the validation group were 0.756 (95% CI 0.516–0.997), 0.759 (95% CI 0.589–0.930), and 0.661 (95% CI 0.430–0.892). The 2-, 3-, and 5-year mean tAUCs of the combined model determined by fivefold cross-validation with 200 repetitions were 0.813 ± 0.123, 0.821 ± 0.085, and 0.770 ± 0.107**,** respectively (Fig. 3C).

The likelihood-ratio test demonstrated that the combined model had better goodness of fit than the clinical and radiomic models in the training cohort but not in the validation cohort (Table 4). The AIC revealed better goodness of fit of the radiomic models than the clinical models in the training and validation cohorts, with an AIC of 356.65 versus 385.53 and an AIC of 76.34 versus 79.24, respectively.

**Table 4:** C-index of the models

Models	C-index (95% CI) Training cohort	*P* value	C-index (95% CI) Validation cohort	*P* value
Clinical model	0.672 (0.599–0.745)	5.422 × 10^–10a^	0.634 (0.593–0.675)	0.085^a^
Radiomics model	0.788 (0.724–0.852)	2.383 × 10^–4a^	0.753 (0.604–0.902)	0.700^a^
Combined model	0.834 (0.777–0.891)	Reference	0.734 (0.688–0.780)	Reference

^a^*P* values calculated by the likelihood ratio-test

### Risk stratification of patients’ OS using the radiomic model

**Fig. 7:**
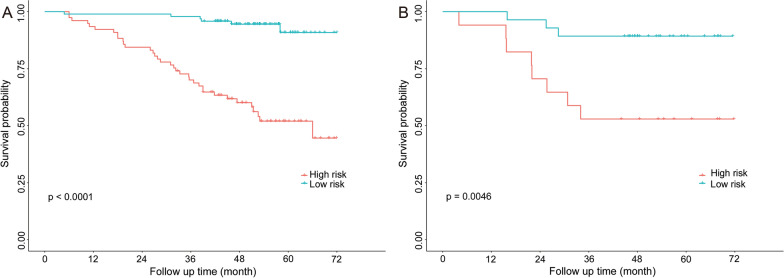
Kaplan–Meier curves of overall survival for stratified analysis of the training cohort (**A**) and validation cohort (**B**). In both training and validation cohorts, patients in the high-risk group had a significantly lower overall survival rate than those in the low-risk group (*p* < 0.01; log-rank test)

Patients were divided into high- and low-risk groups according to the radscore cutoff (0.062261) obtained from the training cohort with maximally selected test statistics. Patients in the high-risk group had a significantly lower OS rate than those in the low-risk group in the training and validation cohorts (*p* < 0.01) (Fig. 7).

## Discussion

In this study, we developed and validated a radiomic signature model based on pretreatment MRI for predicting OS for patients with local residual NPC after IMRT. The radiomic model demonstrated better discrimination and clinical usefulness compared with the clinical model. Additionally, the radiomic model enabled effective risk stratification of patients with local residual disease. The radiomic model may be a low-cost, non-invasive prognostic tool for patients with local residual NPC after IMRT and help select candidates for subsequent treatment.

In our study, the T category, N category and stage were not independent predictors of OS in patients with local residual NPC. It may be related to the fact that patients with local residual tumors after IMRT are mainly characterized by advanced T category and advanced clinical stage. In our cohort, 91.3% of patients had T3 and T4 categories, and 94.0% were locally advanced (stage III and IVa).

In this study, higher age was an adverse prognostic factor in patients with residual NPC after IMRT. For the entire cohort, the 3-year OS of the age < 60 group was about 85.9%, and the 5-year OS was about 78.3%; the corresponding OS of the age ≥ 60 group was about 66.7% and 45.5%, respectively. Previous studies have shown that age is an independent predictor of survival in patients with NPC [24]. Treatment of elderly NPC patients is often more challenging than for younger patients. One of the most significant reasons is that elderly individuals are less likely to tolerate chemotherapy for NPC than younger individuals. In addition, elderly patients are more likely to suffer from comorbid conditions, undoubtedly affecting their survival [25].

This study has also shown that male patients with residual disease after IMRT were associated with poor prognosis. The reasons for male gender as an adverse prognostic factor in patients with residual disease after IMRT may be multifactorial. Male patients are more likely to smoke, and previous studies have demonstrated that smoking has a negative impact on the prognosis of men with NPC [26]. Ouyang et al. used the propensity score matching analysis to investigate the effect of female gender on the prognosis of NPC. After excluding the effects of smoking, tobacco, alcohol, and BMI, female patients still had a better prognosis than male patients. They suggested that the estrogen receptor hypothesis might be one of the reasons for the prognostic difference between female and male patients [27].

In this study, pretreatment albumin level was an independent predictor of OS. A previous study by Shin et al. has demonstrated that baseline albumin levels are a strong prognostic factor in patients with advanced head and neck cancer receiving concurrent chemoradiotherapy [28]. The higher pretreatment albumin levels may be related to better nutritional status and organ function for the patients.

Five radiomic features were included in the radiomic model in our study, with the wavelet filter and the original without filter supplying the most information (n = 2 each), followed by squareroot (n = 1). The features extracted from the wavelet filter may reflect more prognostic information in our study, similar to a previous MRI-based radiomics study [29]. The wavelet features provide the multi-frequency information about the tumor in its multiple dimensions. Original features reflects the information of the tumor on the original image. Squareroot features reflects the square root of the absolute image intensities. Two features belong to the GLDM class in our study. GLDM quantifies gray level dependencies in an image, which is defined as the number of connected voxels within a specified distance that are dependent on the center voxel.

The radiomic signature showed better predictive performance than the clinical model. It may be that radiomics can provide better insight into the heterogeneity of tumors. The heterogeneity of tumors poses a significant challenge in tumor treatment and prognosis. This phenomenon exists between cancers in different patients (intertumoural heterogeneity) as well as within a single tumor (intratumoural heterogeneity) [30]. Intratumoral heterogeneity is characterized by temporal and spatial heterogeneity [31]. The delineation of ROIs in our study was based on the entire tumor, and radiomics, which converts medical images into data that can be mined, may provide more information about the heterogeneity of the whole tumor [32]. Although the C-index of the combined model did not significantly improve over the clinical model in the validation cohort, the decision curve analysis showed better usefulness of the combined model at specific threshold probability. To the best of our knowledge, this is the first study to evaluate the role of MRI-based radiomics in predicting the OS of NPC patients with local residual disease after IMRT.

This study has several limitations: Firstly, although the robustness and avoidance of overfitting of the models were verified in the internal validation cohort, the results should be further validated with multi-center data. Secondly, the follow-up period of the subjects in this study was not long enough, which may impact the assessment of the 5-year prognosis. Thirdly, EBV-DNA copies at diagnosis and the end of chemoradiotherapy have been shown to be a strong prognosis predictor of NPC patients [33]. However, the data of EBV-DNA copies were unavailable during the hospitalization of this series of cases in our hospital, which may affect the models' performance. Fourthly, the diagnosis of local residual tumor was based on MRI findings. False-positive cases may not be completely avoided. For suspected residual lesions without biopsy, the combination of MRI findings, a multidisciplinary setting, endoscopic findings, and Epstein–Barr virus viral load may improve confidence in the diagnosis of residual disease [34].

In conclusion, our study demonstrated that the pretreatment MRI-based radiomic signature model revealed superior performance in predicting the OS of NPC patients with residual disease after IMRT than the clinical model. The radiomic model could be used to stratify patients into high- and low-risk groups, potentially providing additional prognostic information for NPC patients with residual disease after IMRT and potentially helping clinicians personalize medical interventions for patients, thereby improving patient prognosis.

## Supplementary Information

**Additional file 1.** Radiotherapy protocol

**Additional file 2.** Radscore and outcome for each patient

**Additional file 3.** Factors of the combined model predicting OS in patients with local residual tumors after IMRT in the training cohort

## Data Availability

The data used during this study are available from the corresponding author on reasonable request.

## Abbreviations

IMRT: Intensity-modulated radiotherapy
OS: Overall survival
NPC: Nasopharyngeal carcinoma
C-index: Harrell’s concordance index (C-index)
MRI: Magnetic resonance imaging
FRFSE: Fast relaxation fast spin echo
CE-T1WI: Contrast-enhanced T1WI
ROC: Receiver operating characteristic
AUC: Area under curve
AJCC: The American Joint Committee on Cancer
NLR: Neutrophil to lymphocyte ratio
NGTDM: Neighbouring Gray Tone Difference Matrix
GLCM: Gray Level Co-occurrence Matrix
GLDM: Gray Level Dependence Matrix
GLRLM: Gray Level Run Length Matrix
GLSZM: Gray Level Size Zone Matrix
