# Supplementary material for: Development and validation of an MRI-based radiomic model for predicting overall survival in nasopharyngeal carcinoma patients with local residual tumors after intensity-modulated radiotherapy
Source: BMC Med Imaging. 2022 Oct 4;22:174. doi: 10.1186/s12880-022-00902-6 (PMC9533536; doi:10.1186/s12880-022-00902-6)
Supplement: Supplementary file 1 — Additional file 1. Radiotherapy protocol [file 12880_2022_902_MOESM1_ESM.docx]

**Radiotherapy protocol**

Contrast-enhanced CT localization with 3 mm slice thickness ranging from the vertex to 2 cm below the sternal notch was performed. The gross tumor volume of nasopharyngeal carcinoma (GTVnx) and the gross tumor volume of the cervical node (GTVnd) were determined via MRI, endoscopy, and other clinical examinations. The clinical target volume-1 (CTV1) was defined as GTVnx with 5 mm of surrounding expansion. Clinical target volume-2 (CTV2) included CTV1 and 5 mm of surrounding expansion, GTVnd, and potential cervical lymph node metastatic areas. Dosages for primary and subclinical nasopharyngeal lesions were as follows: PTV-GTVnx 68–76 Gy/30–33 f, PTV-CTV1 60–64 Gy/30–33 f, and PTV-CTV2 50–54 Gy/30–33 f; 2.00–2.33 Gy per fraction. The doses administered in the cervical lymph nodes and lymphatic drainage areas were PTV-GTVnd 66-70 Gy/30-33 f and PTV-CTV2 50-54 Gy/30-33 f. Intensity-modulated radiotherapy (IMRT) was performed using Varian clinac iX-SN4948 linear accelerator.
