# Supplementary material for: Development and validation of an MRI-based radiomic model for predicting overall survival in nasopharyngeal carcinoma patients with local residual tumors after intensity-modulated radiotherapy
Source: BMC Med Imaging. 2022 Oct 4;22:174. doi: 10.1186/s12880-022-00902-6 (PMC9533536; doi:10.1186/s12880-022-00902-6)
Supplement: Supplementary file 3 — Additional file 3. Factors of the combined model predicting OS in patients with local residual tumors after IMRT in the training cohort [file 12880_2022_902_MOESM3_ESM.docx]

Factors of the combined model predicting OS in patients with local residual tumors after IMRT in the training cohort

| Characteristics | Value | HR (95% CI) | *P* value |
| --- | --- | --- | --- |
| Age (years) | 45.9 ± 11.6 |  |  |
| ≥ 60 | 20 (11.6%) | 2.230 (1.098-4.532) | 0.027^a^ |
| < 60 | 153 (88.4%) | Reference |  |
| Gender |  |  |  |
| Male | 127 (73.4%) | 2.086 (0.807-5.391) | 0.129^a^ |
| Female | 46 (26.6%) | Reference |  |
| Albumin (g/L) | 43.8 ± 3.5 |  |  |
| ≤ 40.5 | 35 (20.2%) | Reference |  |
| > 40.5 | 138 (79.8%) | 0.354 (0.180-0.695) | 0.003^a^ |
| Radscore | 0.000 ± 0.523 | 12.034 (5.486-26.403) | <0.001^a^ |

^a^*P* values calculated by the multivariate Cox regression analysis.
